# Eye diseases in hot climates (5th edition)

**Published:** 2015

**Figure:**
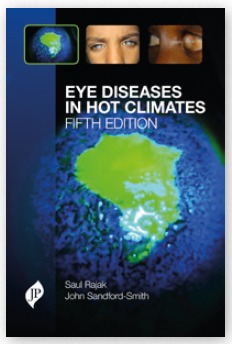


It is now almost 30 years since this excel lent text was first published and ten years since the 4th edition. The book has become a staple for all eye health professionals who have worked in places where blindness is still such a scourge for poor communities.

The 5th edition, with its striking cover illustration, has been extensively revised to reflect the changes that have taken place over the last ten years. Diseases such as macular degeneration and diabetic retinopathy now feature more prominently, but all the main causes of blindness, including cataract, are covered.

The book starts with an excellent review of global blindness, its propensity for hot climates, the impact of VISION 2020 and the community eye health approach to prevention and health education.

One of the pillars of VISION 2020 is the eye care team and this book caters for ophthalmic nurses, clinical officers, assistants and medical students as well as ophthalmologists who will find it an invaluable aid to their practice.

I recommend this book to all those interested in ‘tropical’ ophthalmology and who want a clearly written, beautifully illustrated and comprehensive text.

Nick Astbury

The book is available at the greatly discounted price of £12 + postage and packing (£30 on Amazon) through the Ulverscroft Foundation. To order the book at this price, contact Joyce Sumner, Ulverscroft Foundation, The Green, Bradgate Road, Anstey, Leicestershire, LE7 7FU, UK. tel: +44(0)116 236 1595 fax: +44(0)116 236 1594. Email **foundation@ulverscroft.co.uk** or visit **www.foundation.ulverscroft.com/EyeBooks.htm** to find out about other textbooks available from Ulverscroft.

